# Synthesis, SAR and in vitro therapeutic potentials of thiazolidine-2,4-diones

**DOI:** 10.1186/s13065-018-0496-0

**Published:** 2018-12-04

**Authors:** Sucheta, Sumit Tahlan, Prabhakar Kumar Verma

**Affiliations:** Department of Pharmaceutical Sciences, Maharshi Dayanand University, Rohtak, 124001 India

**Keywords:** Thiazolidine-2,4-dione derivatives, Antimicrobial, antioxidant and antidiabetic activities

## Abstract

**Background:**

Thiazolidinedione is a pentacyclic moiety having five membered unsaturated ring system composed with carbon, oxygen, nitrogen and sulfur molecules at 1 and 3 position of the thiazole ring and widely found throughout nature in various form. They favourably alter concentration of the hormones secreted by adipocytes, particularly adiponectin. They also increase total body fat and have mixed effects on circulating lipids. Thiazolidinedione nucleus is present in numerous biological moieties and has different pharmacological activities likes, e.g. antimalarial, antimicrobial, antimycobacterial, anticonvulsant, antiviral, anticancer, anti-inflammatory, antioxidant, anti-HIV (human immunodeficiency virus) and antituberculosis.

**Results and discussion:**

The synthesized compounds were screened for their in vitro antimicrobial potential against Gram (positive and negative) bacterial and fungal strains by tube dilution technique. In this series, compound **10** exhibited significant antimicrobial activity against *B*. *subtilis* and *S*. *aureus* with MIC = 4.2 × 10^−2^ µM/ml, compound **15** showed significant activity against *K*. *pneumonia* with MIC = 2.60 × 10^−2^ µM/ml and compound **4** displayed potent antibacterial activity against *E*. *coli* with MIC = 4.5 × 10^−2^ µM/ml. Compound **10** had most potent antifungal activity against *C*. *albicans* and *A*. *niger* with MIC = 4.2 × 10^−2^ µM/ml. Compounds **12** and **15** were found as most active antidiabetic agents having IC_50_ = 27.63 μg/ml and 22.35 μg/ml, respectively, using DPPH assay. Antioxidant activity results indicated that compounds **3** and **9** displayed good antioxidant agent with IC_50_ = 29.04 μg/ml and 27.66 μg/ml respectively, using *α amylase* assay.

**Conclusion:**

All the synthesized derivatives exhibited good antimicrobial, antidiabetic and antioxidant activities using specific methods then compared with mentioned standard drugs. Especially, compounds **3**, **4**, **9**, **10**, **12** and **15** displayed highest activity. Structure activity relationship demonstrated that presence of electron withdrawing group (*o*-NO_2_, *p*-Cl, *p*-Br) enhanced the antibacterial activity against *E. coli* as well as increased the antioxidant activity while the presence of electron releasing group (*o/p*-OCH_3_, 3,4,5-trimethoxy) enhanced the antibacterial activity against *S. aureus*, *B. subtilis*, *S*. *typhi*, *K*. *pneumonia*, *C*. *albicans* and *A*. *niger* as well as the antidiabetic activity.

## Background

Thiazolidinedione is a pentacyclic moiety having five membered unsaturated ring system composed with carbon, oxygen, nitrogen and sulfur molecules at 1 and 3 position of the thiazole ring [1]. Thiazolidinedione nucleus is widely found throughout the nature in various forms and have biological activities like antidiabetic [2], antitubercular [3, 4], anticancer activity [5], antimicrobial [6], antioxidant [7] and anti-inflammatory [8]. However, owing to the swift development of new molecules containing this nucleus many research reports have been generated in a brief span of time. Therefore, seems to be requirement to collect recent information in order to understand the current status of the thiazolidinedione nucleus in medicinal chemistry research and specially focuses on the numerous attempts to synthesized and investigate new derivatives with more effective activity [9].

In the last decay, increased resistance of microorganism towards present antimicrobial drugs become a serious problem, that’s why there is a huge requirement of safe, potent and new antimicrobial drugs. Antimicrobial resistance refers to the microorganisms that developed the ability to prohibit, inactivate or block the inhibitory or lethal effects of antimicrobial agents. The antimicrobial resistance towards Gram-positive and Gram-negative strain caused life-threatening infectious diseases in many countries [10]. Antimicrobial drugs are the most powerful incentives in preventing the disease caused by bacteria [9]. The number of antimicrobial drugs available in the market is vast, but there is a need to discover novel antimicrobial agents with better pharmacodynamic and pharmacokinetic properties with lesser or no side effects. Most of thiazolidinediones exhibit good bactericidal activity against various Gram-positive and Gram-negative microbial species. The bactericidal activity of thiazolidinediones derivatives depends on the nature of substitution on the heterocyclic thiazolidine ring rather than the aromatic moiety [11].

Diabetes is a major health problem today, as approximately 5% of the world’s population suffers from diabetes. Type I is prevalent in 10% of diabetes patients and an autoimmune disease of the pancreas, which causes decreased insulin secretion. On the other hand, Type II is prevalent in 90% of the patients where insulin resistance and abnormal carbohydrate metabolism are considered to be the causative [12]. For example, International Diabetes Federation calculated that 4.9 million people deaths over worldwide are due to diabetes, using modeling to calculate the total amount of deaths that could be directly or indirectly attributed to diabetes. Diabetes mellitus occurs throughout the world but it commonly (especially Type 2) occur in more developed countries. The increase in the rate in developing countries follow the trend of urbanization and lifestyle changes, including increasingly sedentary lifestyle, less physically demanding work and the global nutrition transition, marked by increasing intake of foods that are high energy-dense but nutrient-poor (often high in sugar and saturated fats, something referred to as the “western-style” diet) [13].

Thiazolidinediones and their derivatives recently attracted the attention of researchers in exploring their potential as antioxidant agents [7]. Oxidative stress seems to play an important role in many diseases, including cancers. The use of antioxidants in pharmacology is intensively studied, particularly for stroke and neurodegenerative disease [14]. Oxidation of food either by free radicals or by atmospheric oxygen is a serious procedure, which causes the loss of nutritional values and changes in chemical composition. Main function of antioxidant is to neutralize free radicals, which scavenge reactive oxygen species that causes oxidative disease like, neurovascular, autoimmune and cardiovascular disease [7].

**Fig. 1:**
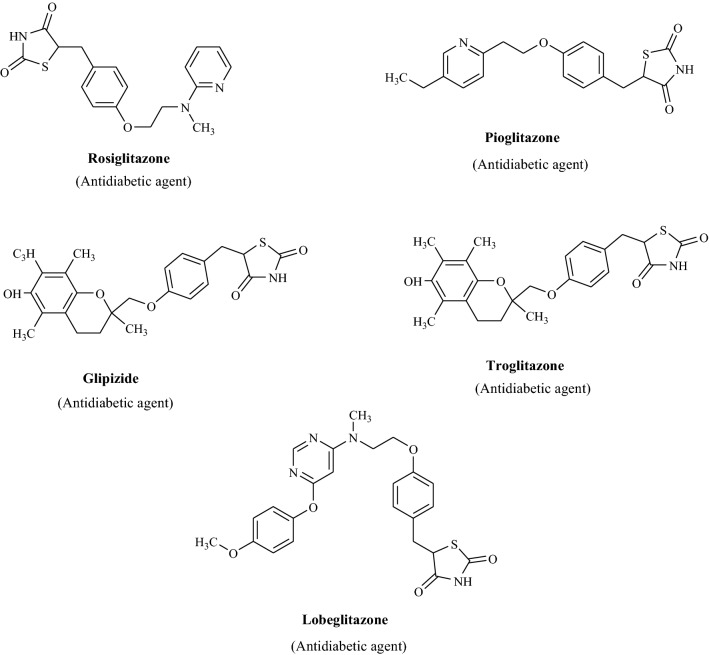
Marketed drugs having thiazolidinedione moiety

Many of the approved drugs having thiazolidinedione moiety are available in commercial market, some of them are given in Fig. 1. Owing to the pharmacological significance of thiazolidinediones derivatives, we have planned to synthesize different biologically active scaffolds of thiazolidinediones followed by their in vitro antimicrobial, antidiabetic and antioxidant activities.

## Results and discussion

### Chemistry

**Scheme 1:**
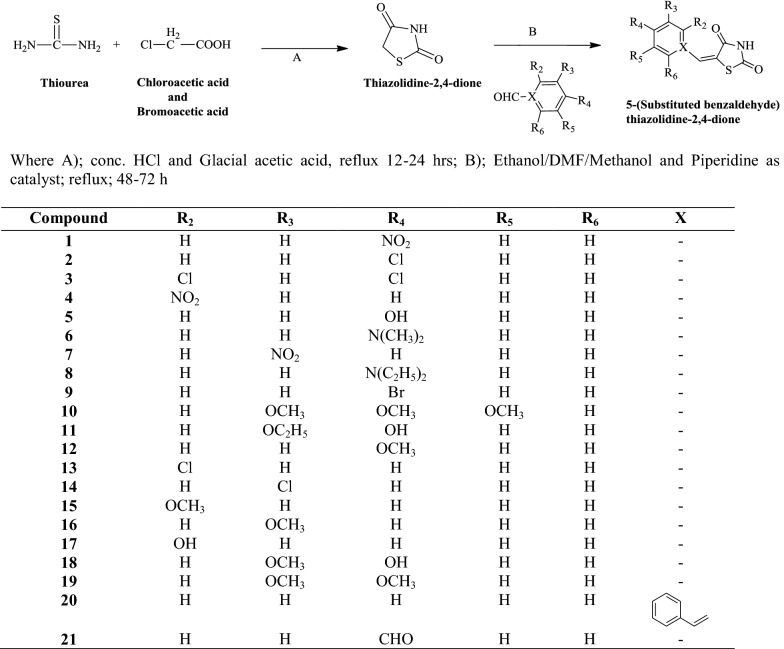
Synthesis of 5-(substituted benzaldehyde)thiazolidine-2,4-diones

In this research work, we synthesized a new series of 5-(substituted benzaldehyde) thiazolidine-2,4-dione analogues using the Knovengeal condensation and the synthetic steps are showed in Scheme 1. The physicochemical properties (molecular formula; molecular weight; melting points; percentage yield and *R*_*f*_ value etc.) of the synthesized analogues are presented in Table 1. The chemical structures of the synthesized derivatives were confirmed by ^1^H/^13^C-NMR, FT-IR and Mass spectrometry.

**Table 1:** The physicochemical properties of newly synthesized derivatives **(1**–**21)**

Compound	M. formula	M. weight	m.pt. (°C)	*R*_*f*_ value	% yield
1	C_10_H_6_N_2_O_4_S	250.23	178–180	0.73	98.00
2	C_10_H_6_ClNO_2_S	239.68	150–153	0.51	98.17
3	C_10_H_5_Cl_2_NO_2_S	274.12	167–170	0.58	95.57
4	C_10_H_6_N_2_O_4_S	250.23	128–130	0.66	98.73
5	C_10_H_7_NO_3_S	221.23	160–165	0.65	97.56
6	C_12_H_12_N_2_O_2_S	248.3	147–150	0.63	79.92
7	C_10_H_6_N_2_O_4_S	250.23	110–113	0.72	95.73
8	C_14_H_16_N_2_O_2_S	276.35	158–160	0.61	67.93
9	C_10_H_6_BrNO_2_S	284.13	150–152	0.63	96.52
10	C_13_H_13_NO_5_S	295.31	210–213	0.61	98.18
11	C_12_H_11_NO_4_S	265.29	158–160	0.72	94.33
12	C_11_H_9_NO_3_S	235.26	187–190	0.71	98.00
13	C_10_H_6_ClNO_2_S	239.68	133–135	0.66	78.89
14	C_10_H_6_ClNO_2_S	239.65	137–140	0.76	88.91
15	C_11_H_9_NO_3_S	235.26	147–150	0.63	77.89
16	C_11_H_9_NO_3_S	235.26	217–220	0.41	95.78
17	C_10_H_7_NO_3_S	221.23	227–230	0.46	89.45
18	C_11_H_9_NO_4_S	251.26	240–243	0.33	97.97
19	C_12_H_11_NO_4_S	265.29	220–223	0.57	81.47
20	C_12_H_9_NO_2_S	231.27	130–133	0.43	90.20
21	C_11_H_7_NO_3_S	233.24	147–150	0.91	96.78

TLC mobile phase-*n*-hexane:ethylacetate

The peak of NO_2_ in compounds **1**, **4** and **7** was found around 1407, 1503 and 1349 cm^−1^. The peak of Cl in compounds **2**, **3**, **13** and **14** was found around 764, 863, 745 and 777 cm^−1^. Compounds **5**, **11**, **17** and **18** has peak of –OH group around 3316, 3405, 3410 and 3459 cm^−1^. Compound **6** has peak of –N(CH_3_)_2_ around 2877 cm^−1^. Compound **8** has peak of –N(C_2_H_5_)_2_ around 1271 cm^−1^. The peak of Br in compound **9** was found around 695 cm^−1^. The peak of –OCH_3_ in compounds **10**, **12**, **15**, **16**, **17**, **18** and **19** was found around 2824, 2835, 2838, 2832, 2842 and 2840 cm^−1^. Compound **11** has found peak of –OC_2_H_5_ around 2871 cm^−1^. The –CHO band in compound **21** was found around 2256 cm^−1^. The ^1^H-NMR multiplet of aromatic benzene was found in between 6.22 and 7.999 δ ppm. The singlet of amine was around 7.896–8.973 δ ppm. Compounds **5**, **11**, **17** and **18** (–OH functional group) have singlet at 2.10–2.67 δ ppm.

The –OCH_3_ singlet (compounds **10**, **12**, **15**, **16**, **18** and **19**) was found in between 3.06 and 3.931 δ ppm. The multiplet of –N(CH_3_)_2_ (compound **6**) was found around 8.256–8.694 δ ppm. Compound **8** have multiplet of –N(C_2_H_5_)_2_ at 8.44–8.58 δ ppm. The singlet of –CHO (compound **21**) was found 10.16 δ ppm.

#### Antimicrobial activity

The in vitro antimicrobial activity of synthesized compounds was done by tube dilution method against tested microorganisms. In case of Gram positive bacteria, compound **10** (MIC_*sa*, *bs*_ = 4.2 × 10^−2^ µM/ml) found most active against *S. aureus* and *B*. *subtilis* while in case of Gram negative bacteria, compound **15** (MIC_*kp*_ = 2.60 × 10^−2^ µM/ml) had most potent activity against *K*. *pneumonia* while compound **4** (MIC_*ec*_ = 4.5 × 10^−2^ µM/ml) was found active against *E*. *coli*. Antifungal activity results revealed that compound **10** (MIC_*ca* & *an*_ = 4.2 × 10^−2^ µM/ml) displayed as most potent antifungal agent against *C. albicans* and *A. niger*. These compounds may be taken as lead to discovery novel antimicrobial agents. The presented results are showing in Table 2.

**Table 2:** Antimicrobial activity (MIC = μM/ml) of newly synthesized compounds

Compound	Minimum inhibitory concentration (μΜ/ml)						
	Bacteria					Fungi	
	*B*. *subtilis*	*S*. *aureus*	*K*. *pneumonia*	*E*. *coli*	*S*. *typhi*	*A*. *niger*	*C*. *albicans*
1	9.90	4.90	9.90	4.90	4.90	4.90	4.90
2	10.40	5.21	5.21	5.21	20.80	5.21	5.21
3	4.50	4.50	9.12	9.12	4.50	4.50	4.50
*4*	9.90	4.99	9.90	*4.50*	4.99	4.99	4.99
5	5.65	5.65	11.30	11.30	5.65	5.65	5.65
6	5.00	5.00	10.06	4.99	5.00	10.06	20.10
7	9.90	4.99	4.99	4.99	4.99	9.90	4.99
8	4.50	4.50	4.50	5.00	4.50	4.50	4.50
9	8.79	4.30	8.79	8.79	4.30	4.30	4.30
*10*	*4.20*	*4.20*	4.20	8.40	*4.20*	*4.20*	*4.20*
11	9.40	4.70	4.70	4.70	4.70	4.70	4.70
12	10.60	10.60	10.60	5.31	5.31	5.31	5.31
13	10.40	5.21	10.40	5.21	5.21	2.60	5.21
14	5.21	5.21	10.60	10.40	5.21	2.60	5.21
*15*	5.31	5.31	*2.60*	5.31	5.31	5.31	5.31
16	5.31	5.31	10.60	5.31	5.31	5.31	5.31
17	11.30	5.65	11.30	5.65	5.65	5.65	5.65
18	4.90	4.90	9.90	4.90	4.90	4.90	4.90
19	4.70	4.70	9.40	4.70	4.70	4.70	4.70
20	5.40	5.40	10.80	5.40	5.40	5.40	5.40
21	5.30	5.30	10.70	5.30	5.30	5.30	5.30
Cefadroxil	3.40	1.71	3.40	1.71	1.71	–	–
Fluconazole	–	–	–	–	–	4.08	4.08

Compound numbers and their significant values are given in italic

#### Antidiabetic activity

**Fig. 2:**
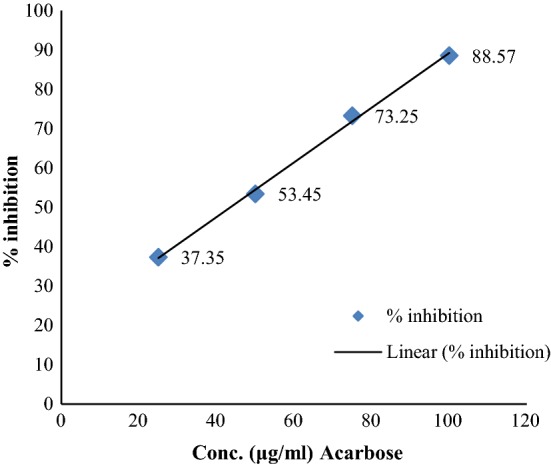
Standard graph of acarbose

**Fig. 3:**
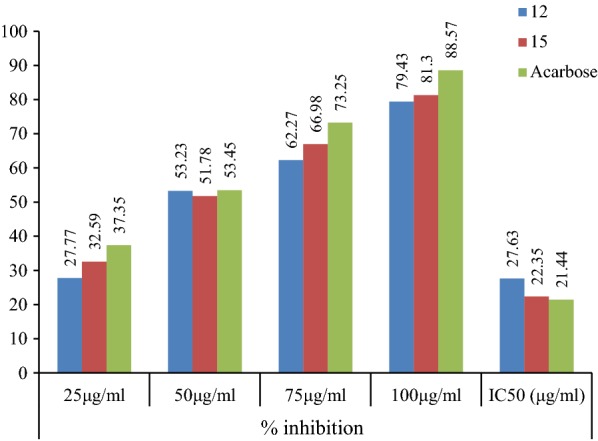
Graph of potent antidiabetic compounds 12 and 15

**Fig. 4:**
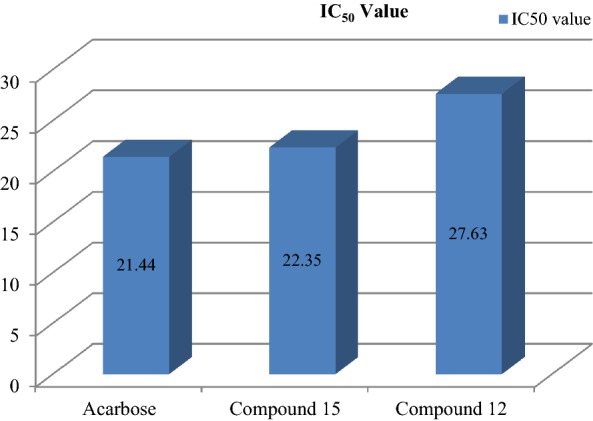
IC_50_ values of compounds 12 and 15 compared with acarbose

The results of antidiabetic activity showed that few of synthesized compounds exhibited considerable antidiabetic activity while other showed good to moderate antidiabetic activity. In this series, only compounds **12** and **15** exhibited excellent antidiabetic activity with IC_50_ value of 27.63 and 22.35 μg/ml (Table 3). The IC_50_ value was calculated via the graph plotted between % inhibition and compound (Figs. 2, 3 and 4).

**Table 3:** Antidiabetic activity of synthesized compounds

Compound	% inhibition				
	25 μg/ml	50 μg/ml	75 μg/ml	100 μg/ml	IC_50_ (μg/ml)
1	22.65	35.79	55.52	88.68	54.35
2	17.89	42.62	59.78	74.67	47.51
3	29.45	47.98	60.83	95.79	43.45
4	31.26	40.65	63.93	92.61	43.63
5	18.26	47.61	49.24	96.72	56.64
6	33.56	49.45	50.78	97.15	37.35
7	24.72	35.71	64.87	97.15	56.09
8	20.79	39.54	40.89	93.17	56.89
9	24.73	53.78	68.34	89.37	41.67
10	29.34	43.98	63.36	98.14	47.49
11	18.49	47.87	59.74	89.71	52.69
*12*	27.77	53.23	62.27	79.43	*27.63*
13	25.74	43.89	56.76	92.72	48.45
14	21.78	48.82	72.54	93.92	50.80
*15*	32.59	51.78	66.98	81.30	*22.35*
16	27.52	43.73	57.27	85.23	40.92
17	23.67	48.56	65.46	93.17	48.48
18	18.64	45.85	65.11	95.98	56.39
19	29.33	47.77	58.34	90.86	40.01
20	34.73	45.98	68.34	88.45	31.62
21	21.98	47.28	70.56	74.76	38.65
Acarabose	37.35	53.45	73.25	88.57	21.44

Compound numbers and their significant values are given in italic

#### Antioxidant activity

**Fig. 5:**
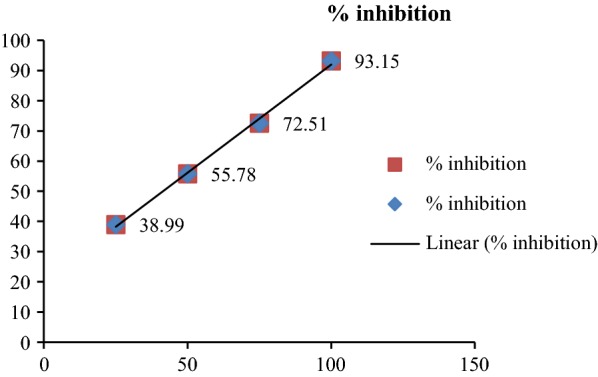
Standard graph of ascorbic acid

**Fig. 6:**
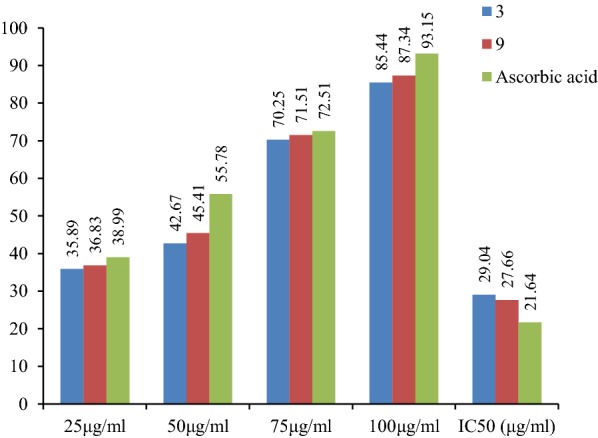
Graph of potent antioxidant compound 3 and 9

**Fig. 7:**
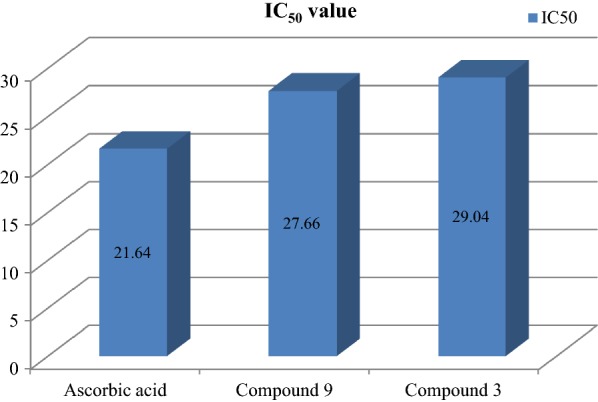
IC_50_values of compound 3 and 9 compared to ascorbic acid

The results of antioxidant activity showed that few of synthesized derivatives showed considerable antioxidant activity while the other showed good to moderate antioxidant activity. Among them, compounds **3** and **19** exhibited excellent antioxidant activity (IC_50_ = 29.04 and 27.66 μg/ml), respectively. The presented results are showing in Table 4. The IC_50_ value was calculated via the graph plotted between % inhibition and compound (Figs. 5, 6 and 7).

**Table 4:** Antioxidant activity of newly synthesized derivatives

Compound	% inhibition				
	25 μg/ml	50 μg/ml	75 μg/ml	100 μg/ml	IC_50_ (μg/ml)
1	32.64	40.22	73.41	84.81	36.35
2	15.98	35.41	74.05	93.79	62.14
*3*	35.89	42.67	70.25	85.44	*29.04*
4	29.45	45.75	79.74	83.10	36.17
5	35.79	39.78	62.02	85.47	31.10
6	19.54	38.93	62.65	75.82	48.13
7	20.51	35.61	79.74	86.04	55.23
8	26.93	33.78	62.23	65.78	31.15
*9*	36.83	45.41	71.51	87.34	*27.66*
10	28.61	39.67	59.75	86.70	43.44
11	18.64	25.71	72.78	93.59	64.03
12	20.67	42.75	77.84	90.50	53.27
13	24.45	44.76	71.51	77.84	39.41
14	29.81	39.78	65.18	95.18	47.61
15	33.51	43.98	77.21	98.67	43.25
16	30.65	43.62	79.11	87.97	39.77
17	32.78	40.24	69.55	89.87	40.06
18	36.94	43.52	60.12	93.03	33.51
19	28.64	39.67	73.41	90.05	46.03
20	25.64	46.93	69.55	87.97	43.89
21	17.91	41.64	75.98	92.32	55.51
Ascorbic acid	38.99	55.78	72.51	93.15	21.64

Compound numbers and their significant values are given in italic

#### SAR (structure activity relationship) studies

**Fig. 8:**
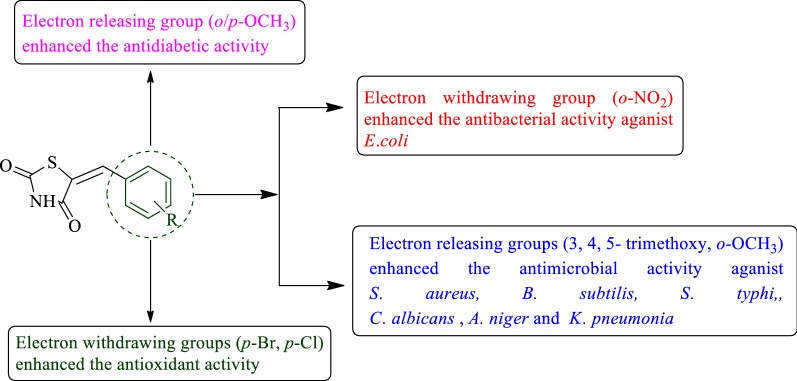
Structural activity relationship studies of synthesized derivatives

From the antimicrobial, antidiabetic and antioxidant activities results of newly synthesized 5-(substituted benzaldehyde) thiazolidine-2,4-dione derivatives, the consequently structure activity relationship can be derived (Fig. 8): presence of electron releasing group (3,4,5-trimethoxy, *p/o*-OCH_3_, compounds **10**, **12** and **15**) on benzylidene portion of thiazolidinedione improved antimicrobial against *S*. *aureus*, *B*. *subtilis*, *S*. *typhi*, *C*. *albicans, A*. *niger*, *K*. *pneumonia* and antidiabetic activity of the synthesized derivatives respectively. Presence of electron withdrawing group (*o*-NO_2_, *p*-Cl, *p*-Br, compounds **4**, **3**, and **9**) on benzylidene portion of thiazolidinedione improved the antibacterial activity against *E*. *coli* and enhanced the antioxidant activity of the synthesized compounds respectively. From these result we may conclude that different structural requirements are required for a compound to be effective against different targets.

#### Experimental section

Synthesized thiazolidine-2,4-diones derivatives followed the general procedure as discussed in Scheme 1. All reagents and solvents used in the study were of both laboratory and analytical grade. Reaction steps forward was observed by thin layer chromatography (TLC) making use of commercial silica gel plates. Melting points were done in open capillary tubes method. ^1^H/^13^C-NMR spectra were recorded by Bruker Avance 400 NMR spectrometer in CDCl_3_-deuterated solvent and expressed in parts per million (δ, ppm) downfield from tetramethyl silane (internal standard). ^1^H-NMR data are given as multiplicity (s, singlet; d, douplet; t, triplet; m, multiplet) and number of proton. Infrared (IR) spectrum was recorded on a Bruker FTIR 12060280, Software: OPUS 7.2.139.1294 spectrophotometer. Waters Micromass Q-ToF Micro instrument was used for obtaining the Mass spectra.

#### General procedure for the synthesis of thiazolidine-2,4-diones derivatives (**1**–**21**)

##### *Step*-*a: Synthesis of thiazolidine*-*2,4*-*dione (A)*

Chloroacetic acid (0.1 mol) in 10 ml of water and thiourea (0.1 mol) dissolved in 10 ml of water, both the solution were mixed and stirred for 15 min until white precipitate was obtained then cooled. After that 10 ml hydrochloric acid was added slowly in a reaction mixture with a dropping funnel. The flask was then connected with a reflux condenser and gentle heat applied, after that the reaction mixture was stirred and refluxed for 8–10 h at 100–110 °C. The product was cooled, filtered, washed and dried at room temperature followed by recrystallization with suitable solvent [9].

##### *Step*-*b: Synthesis of N′*-*(Subtituted benzaldyhyde)*-*2,4*-*thiazolidinedione (B)*

The solution of 2,4-thiazolidinedione (A) (0.01 mol) and different benzaldehyde (0.01 mol) was suspended in ethanol/DMF/methanol with catalytic amount of piperidine (1 ml) and mixture was shaken for few minutes and then refluxed for 48–72 h. After that the reaction mixture was cooled at room temperature. The product precipitated out from ethanol and separated by using separating funnel followed by recrystallization with suitable solvent [9].

#### Spectral data of synthesized thiazolidinediones derivatives

FT-IR (KBr pellets, cm^−1^) and ^1^H/^13^C-NMR (CCl_4_, δ ppm), stretching = st.; Exp. = expected; Cal. = calculated.

##### Compound **1**: 5-(4-Nitrobenzylidene)thiazolidine-2,4-dione (IR)

3029 (C–H str., aromatic), 1602 (C=C str., aromatic), 1677 (–CONH str., amide) 1748 (–CO str., carbonyl), 1710 (C=C str., aliphatic), 2590 (S str., thiazole ring) 1407 (C–NO_2_ str., aromatic)); ^13^C-NMR: 116.12, 121.27, 121.57, 127.34, 127.43, 141.32, 147.82, 166.41, 167.21; ^1^H-NMR: 6.457–6.925 (d, 2H, Ar–H), 7.863–7.992 (d, 2H, Ar–H), 8.124–8.56 (m, 2H, Ar–H), 8.763 (s, 1H, NH, amine); MS: *m/z* 261.55 (Exp.), 262.60 (Cal.) [M^+^ +1].

##### Compound **2**: 5-(4-Chlorobenzylidene)thiazolidine-2,4-dione (IR)

3046 (C–H str., aromatic), 1486 (C=C str., aromatic), 1622 (–CONH str., amide), 1704 (–CO str., carbonyl), 1607 (C=C str., aliphatic), 2618 (S str., thiazole ring), 764 (C–Cl str., aromatic)); ^13^C-NMR: 115.98, 127.92, 127.99, 128.85, 129.12, 133.52, 133.62, 166.35,167.21; ^1^H-NMR: 7.364–7.848 (m, 4H, Ar–H), 8.153–8.473 (m, 2H, Ar–H), 8.697 (s, 1H, NH, amine); MS: *m/z* 228.55 (Exp.), 229.53 (Cal.) [M^+^ +1].

##### Compound **3**: 5-(2,4-Dichlorobenzylidene)thiazolidine-2,4-dione (IR)

3009 (C–H str., aromatic), 1437 (C=C str., aromatic), 1697 (–CONH str., amide), 1749 (–CO str., carbonyl), 1574 (C=C str., aliphatic), 2557 (S str., thiazole ring), 863 (C–Cl str., aromatic); ^13^C-NMR: 115.62, 126.52, 129.32, 130.31, 131.22, 132.51, 134.85, 167.21, 166.41; ^1^H-NMR: 7.284–7.550 (m, 5H, Ar–H), 8.142 (s, 1H, NH, amine); MS: *m/z* 261.69 (Exp.), 262.43 (Cal.) [M^+^ +1].

##### Compound **4**: 5-(2-Nitrobenzylidene)thiazolidine-2,4-dione (IR)

3075 (C–H str., aromatic), 1422 (C=C str., aromatic), 1648 (–CONH str., amide) 1741 (–CO str., carbonyl), 1533 (C=C str., aliphatic), 2624 (S str., thiazole ring), 1503 (C–NO_2_ str., aromatic); ^13^C-NMR: 115.51, 121.12, 127.21, 128.81, 130.12, 134.61, 146.21, 166.12, 167.21; ^1^H-NMR: 7.284–7.999 (m, 4H, Ar–H), 8.139–8.23 (m, 2H, Ar–H), 8.396 (s, 1H, NH, amine); MS: *m/z* 228.55 (Exp.), 229.35 (Cal.) [M^+^ +1].

##### Compound **5**: 5-(4-Hydroxybenzylidene)thiazolidine-2,4-dione (IR)

3125 (C–H str., aromatic), 1444 (C=C str., aromatic), 1679 (–CONH str., amide), 1725 (–CO str., carbonyl), 1572 (C=C str., aliphatic), 2555 (S str., thiazole ring), 3316 (–OH str., aromatic)); ^13^C-NMR: 115.31, 115.68, 115.91, 127.25, 127.38, 127.49, 157.25, 166.35, 167.21; ^1^H-NMR: 7.12–7.67 (m, 2H, Ar–H), 8.124–8.792 (m, 4H, Ar–H), 2.67 (s, 1H, OH), 8.898 (s, 1H, NH, amine); MS: *m/z* 253.55 (Exp.), 252.67 (Cal.) [M^+^ +1].

##### Compound **6**: 5-(4-(Dimethylamino)benzylidene)thiazolidine-2,4-dione (IR)

3062 (C–H str., aromatic), 1430 (C=C str., aromatic), 1681 (–CONH str., amide), 1717 (–CO str., carbonyl), 1557 (C=C str., aliphatic), 2551 (S str., thiazole ring), 2877 (C–NH(CH_3_)_2_ str., aromatic)); ^13^C-NMR: 40.51, 40.59, 114.25, 114.31, 115.91, 124.61, 127.35, 127.41, 148.81, 166.35, 167.25; ^1^H-NMR: 6.22–6.53 (m, 2H, Ar–H), 7.135–7.662 (m, 4H, Ar–H), 8.256–8.694 {m, 6H, (CH_3_)_2_}, 8.973 (s, 1H, NH, amine); MS: *m/z* 246.75 (Exp.), 247.78 (Cal.) [M^+^ +1].

##### Compound **7**: 5-(3-Nitrobenzylidene)thiazolidine-2,4-dione (IR)

3141 (C–H str., aromatic), 1526 (C=C str., aromatic), 1689 (–CONH str., amide) 1742 (–CO str., carbonyl), 1597 (C=C str., aliphatic), 2532 (S str., thiazole ring), 1349 (C–NO_2_ str., aromatic)); ^13^C-NMR: 115.92, 120.39, 121.25, 129.69, 132.58, 136.29, 148.39, 166.39, 167.25; ^1^H-NMR: 7.16–7.65 (m, 2H, Ar–H), 7.49 (m, 2d, Ar–H), 7.932–8.095 (m, 2H, Ar–H), 8.335 (s, 1H, NH, amine); MS: *m/z* 223.85 (Exp.), 224.86 (Cal.) [M^+^ +1].

##### Compound **8**: 5-(Diethylamino)benzylidene)thiazolidine-2,4-dione (IR)

3055 (C–H str., aromatic), 1440 (C=C str., aromatic), 1662 (–CONH str., amide), 1715 (–CO str., carbonyl), 1591 (C=C str., aliphatic), 2615 (S str., thiazole ring) (C–N str.,), 1271 (C–N(C_2_H_5_)_2_ str., aromatic)); ^13^C-NMR: 13.25, 4.73, 44.82, 114.25, 114.29, 115.81, 124.81, 127.42, 127.51, 148.88, 166.35, 167.23; ^1^H-NMR: 6.65–7.62 (m, 3H, Ar–H), 8.142 (s, 1H, Ar–H), 7.892–7999 (m, 2H, Ar–H), 8.44–8.58 {m, 10H, (C_2_H_5_)_2_}, 8.793 (s, 1H, NH, amine); MS: *m/z* 252.75 (Exp.), 253.74 (Cal.) [M^+^ +1].

##### Compound **9**: 5-(4-Bromobenzylidene)thiazolidine-2,4-dione (IR)

3169 (C–H str., aromatic), 1482 (C=C str., aromatic), 1651 (–CONH str., amide), 1715 (–CO str., carbonyl), 1591 (C=C str., aliphatic), 2522 (S str., thiazole ring), 695 (C–Br str., aromatic); ^13^C-NMR: 115.95, 122.39, 128.69, 128.71, 131.65, 131.69, 134.31, 166.42, 167.25; ^1^H-NMR: 7.284–7.805 (m, 5H, Ar–H), 8.261 (s, 1H, NH, amine); MS: *m/z* 244.88 (Exp.), 245.89 (Cal.) [M^+^ +1].

##### Compound **10**: 5-(3,4,5-Trimethoxybenzylidene)thiazolidine-2,4-dione (IR)

3127 (C–H str., aromatic), 1456 (C=C str., aromatic), 1692 (–CONH str., amide), 1738 (–CO str., carbonyl), 1594 (C=C str., aliphatic), 2539 (S str., thiazole ring), 2824 (–OCH_3_ str., aromatic); ^13^C-NMR: 50.58, 56.29, 56.32, 103.99, 104.12, 115.99, 129.55, 138.29, 150.75, 150.80, 166.39, 167.18; ^1^H-NMR: 3.942 {s, 9H, (OCH_3_)_3_}, 6.754 (d, 2H, Ar–H), 7.284–7.794 (m, 3H, Ar–H), 7.809 (s, 1H, Ar–H), 8.747 (s, 1H, NH, amine); MS: *m/z* 232.89 (Exp.), 233.88 (Cal.) [M^+^ +1].

##### Compound **11**: 5-(4-Hydroxy-3-ethoxybenzylidene)thiazolidine-2,4-dione (IR)

3141 (C–H str., aromatic), 1442 (C=C str., aromatic), 1586 (–CONH str., amide), 1693 (–CO str., carbonyl), 1510 (C=C str., aliphatic), 2565 (S str., thiazole ring), 2871 (–OC_2_H_5_ str., aromatic), 3405 (OH str., aromatic)); ^13^C-NMR: 56.25, 112.18, 115.88, 116.89, 120.91, 128.88, 144.95, 151.35, 166.35, 167.91; ^1^H-NMR: 7.45–7.49 (m, 2H, Ar–H), 8.284–8.499 (m, 4H, Ar–H), 2.10 (s, 1H, OH), 3.86 (s, 5H, C_2_H_5_), 8.823 (s, 1H, NH, amine); MS: *m/z* 256.65 (Exp.), 257.63 (Cal.) [M^+^ +1].

##### Compound **12**: 5-(4-methoxybenzylidene)thiazolidine-2,4-dione (IR)

3127 (C–H str., aromatic), 1465 (C=C str., aromatic), 1699 (–CONH str., amide) 1733 (–CO str., carbonyl), 1588 (C=C str., aliphatic), 2588 (S str., thiazole ring), 2835 (–OCH_3_ str., aromatic); ^13^C-NMR: 55.99, 114.28, 115.11, 115.93, 127.49, 127.53, 127.63, 160.10, 166.39, 167.27; ^1^H-NMR: 3.899 (s, 3H, OCH_3_, methoxy), 7.025 (d, 2H, Ar–H), 7.455 (d, 2H, Ar–H), 8.322 (s, 1H, NH, amine); MS: *m/z* 284.78 (Exp.), 285.88 (Cal.) [M^+^ +1].

##### Compound **13**: 5-(2-Chlorobenzylidene)thiazolidine-2,4-dione (IR)

3068 (C–H str., aromatic), 1462 (C=C str., aromatic), 1634 (–CONH str., amide), 1734 (–CO str., carbonyl), 1672 (C=C str., aliphatic), 2539 (S str., thiazole ring), 745 (C–Cl str., aromatic)); ^13^C-NMR: 116.12, 126.85, 127.83, 128.88, 131.29, 133.22, 166.35, 167.23; ^1^H-NMR: 7.15–7.59 (m, 2H, Ar–H), 8.043–8.591 (m, 4H, Ar–H), 8.953 (s, 1H, NH, amine); MS: *m/z* 232.65 (Exp.), 238.67 (Cal.) [M^+^ +1].

##### Compound **14**: 5-(3-Chlorobenzylidene)thiazolidiine-2,4-dione (IR)

3046 (C–H str., aromatic), 1470 (C=C str., aromatic), 1699 (–CONH str., amide), 1742 (–CO str., carbonyl), 1612 (C=C str., aliphatic), 2543 (S str., thiazole ring), 777 (C–Cl str., aromatic)); ^13^C-NMR: 115.55, 124.55, 126.57, 128.23, 130.22, 134.26, 136.66, 166.42, 167.22; ^1^H-NMR: 6.324 (d, 2H, Ar–H), 7145–7.873 (m, 3H, Ar–H), 7.939 (s, 1H, Ar–H), 8.363 (s, 1H, NH, amine); MS: *m/z* 243.65 (Exp.), 244.65 (Cal.) [M^+^ +1].

##### Compound **15**: 5-(2-Methoxybenzylidene)thiazolidine-2,4-dione (IR)

3132 (C–H str., aromatic), 1461 (C=C str., aromatic), 1677 (–CONH str., amide), 1738 (–CO str., carbonyl), 1586 (C=C str., aliphatic), 2552 (S str., thiazole ring), 2838 (–OCH_3_ str., aromatic); ^13^C-NMR: 56.35, 114.26, 115.28, 115.98, 121.91, 127.23, 129.42, 157.73, 1666.3, 167.15; ^1^H-NMR: 3.931 (s, 3H, OCH_3_, methoxy), 6.966–7.072 (m, 2H, Ar–H), 7.438–7.448 (m, 2H, Ar–H), 7.284 (s, 1H, Ar–H), 8.263 (s, 1H, NH, amine); MS: *m/z* 252.60 (Exp.), 253.62 (Cal.) [M^+^ +1].

##### Compound **16**: 5-(3-Methoxybenzylidene)thiazolidine-2,4-dione (IR)

3092 (C–H str., aromatic), 1483 (C=C str., aromatic), 1677 (–CONH str., amide), 1727 (–CO str., carbonyl), 1596 (C=C str., aliphatic), 2562 (S str., thiazole ring), 2832 (–OCH_3_ str., aromatic)); ^13^C-NMR: 55.92, 110.62, 113.53, 115.92, 118.73, 129.75, 136.25, 160.63, 166.36, 167.18; ^1^H-NMR: 3.68 (s, 3H, OCH_3_, methoxy), 6.725 (d, 2H, Ar–H), 7.655 (d, 2H, Ar–H), 7.982 (s, 1H, NH, amine); MS: *m/z* 239.65 (Exp.), 240.65 (Cal.) [M^+^ +1].

##### Compound **17**: 5-(2-Hydroxybenzylidene)thiazolidine-2,4-dione (IR)

3112 (C–H str., aromatic), 1454 (C=C str., aromatic), 1671 (–CONH str., amide), 1722 (–CO str., carbonyl), 1590 (C=C str., aliphatic), 2549 (S str., thiazole ring), 3410 (OH str., aromatic)); ^13^C-NMR: 115.82, 115.93, 116.63, 121.33, 127.83, 129.44, 158.33, 166.34, 167.17; ^1^H-NMR: 6.562–6.937 (m, 2H, Ar–H), 7.024–7.792 (m, 4H, Ar–H), 2.32 (s, 1H, OH), 8.364 (s, 1H, NH, amine); MS: *m/z* 294.79 (Exp.), 295.75 (Cal.) [M^+^ +1].

##### Compound **18**: 5-(4-Hydroxy-3-methoxybenzylidene)thiazolidine-2,4-dione (IR)

3140 (C–H str., aromatic), 1449 (C=C str., aromatic), 1680 (–CONH str., amide), 1725 (–CO str., carbonyl), 1578 (C=C str., aliphatic), 2617 (S str., thiazole ring), 2842 (–OCH_3_ str., aromatic), 3459 (OH str., aromatic)); ^13^C-NMR: 56.23, 112.11, 115.92, 116.82, 120.13,128.81, 144.91, 151.33, 166.33, 167.19; ^1^H-NMR: 6.245–6.949 (m, 2H, Ar–H), 7.584–7.949 (m, 4H, Ar–H), 2.54 (s, 1H, OH), 3.06 (s, 3H, OCH_3_), 8.435 (s, 1H, NH, amine); MS: *m/z* 274.85 (Exp.), 275.89 (Cal.) [M^+^ +1].

##### Compound **19**: 5-(3,4-Dimethoxybenzylidene)thiazolidine-2,4-dione (IR)

3072 (C–H str., aromatic), 1459 (C=C str., aromatic), 1658 (–CONH str., amide), 1703 (–CO str., carbonyl), 1624 (C=C str., aliphatic), 2606 (S str., thiazole ring), 2840 (–OCH_3_ str., aromatic)); ^13^C-NMR: 56.22, 56.25, 111.61, 115.25, 115.92, 119.73, 128.51, 149.11, 149.73, 166.32, 167.18; ^1^H-NMR: 3.48 {s, 6H, (OCH_3_)_2_}, 6.135 (d, 2H, Ar–H), 7.024–7.694 (m, 3H, Ar–H), 7.896 (s, 1H, Ar–H), 8.463 (s,1H, NH, amine); MS: *m/z* 234.25 (Exp.), 235.23 (Cal.) [M^+^ +1].

##### Compound **20**: 5-((*E*)-3-Phenylallylidene)thiazolidine-2,4-dione (IR)

3049 (C–H str., aromatic), 1443 (C=C str., aromatic), 1680 (–CONH str., amide), 1723 (–CO str., carbonyl), 1607 (C=C str., aliphatic), 2624 (S str., thiazole ring)); ^13^C-NMR: 119.12, 125.32, 126.42, 126.45, 128.11, 128.74, 128.77, 131.26, 135.25, 136.18, 166.39, 167.15; ^1^H-NMR: 6.424 (d, 2H, Ar–H), 6.745–7.273 (m, 4H, Ar–H), 7.495–7.939 (m, 2H, Ar–H), 8.763 (s, 1H, NH, amine), 6.92 (s, 2H, vinyl proton); MS: *m/z* 253.45 (Exp.), 254.48 (Cal.) [M^+^ +1].

##### Compound **21**: 4-((2,4-Dioxothiazolidin-5-ylidene)methyl)benzaldehyde (IR)

3120 (C–H str., aromatic), 1463 (C=C str., aromatic), 1698 (–CONH str., amide), 1751 (–CO str., carbonyl), 1603 (C=C str., aliphatic), 2541 (S str., thiazole ring), 2256 (CHO str., aromatic); ^13^C-NMR: 115.95, 126.93, 126.98, 129.83, 129.93, 136.15, 141.13, 191.13, 166.33, 167.15; ^1^H-NMR: 6.484–6.999 (m, 4H, Ar–H), 7.139–7.537 (m, 2H, Ar–H), 7.896 (s, 1H, NH, amine), 10.16 (s, 1H, CHO); MS: *m/z* 281.75 (Exp.), 282.72 (Cal.) [M^+^ +1].

### Biological activities

#### Antimicrobial activity

The in vitro antimicrobial activity of synthesized compounds was done by tube dilution method against Gram-positive bacteria: *Staphylococcus aureus*, *Bacillus subtilis,* Gram-negative bacteria: *Escherichia coli*, *Klebsiella pneumonia*, *Salmonella typhi* and fungal: *Candida albicans* and *Aspergillus niger* strains [15] using cefadroxil and fluconazole as standard. Dilutions of test and standard compounds were prepared in double strength nutrient broth for bacterial strains and Sabouraud dextrose broth for fungal strains [16]. The samples were incubated at 37 ± 1 °C for 24 h (for bacterial species), at 25 ± 1 °C for 7 days (*A. niger*) and at 37 ± 1 °C for 48 h (*C. albicans*) respectively and the results were recorded in terms of MIC (the lowest concentration of test substance which inhibited the growth of microorganisms).

#### Antidiabetic activity

All the synthesized compounds were evaluated against *α*-*amylase* inhibitory activity by using *diastase* based on colorimetric method [17]. 0.25 g of soluble potato starch was dissolved in 50 ml of 20 mM phosphate buffer by heating for 15 min. 1 mg *diastase* (*amylase* enzyme) was mixed in 100 ml of 20 mM phosphate buffer (pH 6.9) to obtain the enzyme solution. Different concentrations of all the synthesized derivatives were prepared by dissolving them in DMSO. The color reagent was prepared by mixing 20 ml of 96 mM 3,5-dinitrosalicylic acid with 5.31 M sodium potassium tartrate in 8 ml of 2 M sodium hydroxide and 12 ml deionized water. 1 ml of enzyme solution was mixed with 1 ml of each synthesized derivatives and incubated for 10 min at 25 °C. Then 1 ml of this mixture was mixed with 1 ml of soluble potato starch solution in a tube and incubated for 10 min at 25 °C. Then tubes were closed after adding 1 ml of color reagent and placed into water bath for 15 min at 85 °C. The reaction mixture was removed from water bath after 15 min. After cooling, the reaction mixture was diluted with 9 ml of distilled water and the absorbance was taken at 540 nm in UV spectrophotometer. Blank solution was prepared by replacing the enzyme solution with buffer solution and absorbance was taken. Measurement of control was performed in identical manner by replacing the synthesized derivatives in 1 ml of DMSO. Acarbose solution was used as a standard drug [18].

Percentage inhibition of α-*amylase* enzyme was calculated by using following formula:

1 $$\% \,{\text{Inhibition}}\,{ = }\,\frac{{{\text{A}}_{\text{Blank}} \, - \,{\text{A}}_{\text{Sample}} }}{{{\text{A}}_{\text{Blank}} }}\, \times \,100$$

#### Antioxidant activity

The antioxidant activity of the newly synthesized compounds were evaluated spectrophotometrically using free radical scavenging method by DPPH (2,2-diphenyl-1-picrylhydrazyl) assay. The DPPH is a stable free radical with maximal absorption at 517 nm and is reduced to a corresponding hydrazine when it reacting with hydrogen donors. When DPPH reacts with an antioxidant agent, gets reduced by donating hydrogen and its color change from deep violet to yellow, which shows a considerable decrease in absorption at 517 nm. DPPH solution (3 μg/ml) was prepared in methanol and DPPH (in 1:1) solution was used for blank reference. Four dilutions of different concentrations (25, 50, 75 and 100 μg/ml) of each synthesized compound and standard (ascorbic acid) were prepared in the methanol and 1 ml of each concentration was added to 1 ml of DPPH solution. The solution mixture was shaken vigorously and kept in dark place for 30 min at room temperature and absorbance was measured by UV at 517 nm [19].

Percentage inhibition of Free radical DPPH was calculated as follows:

1 $$\% \,{\text{Inhibition}}\,{ = }\,\frac{{{\text{A}}_{\text{Blank}} \, - \,{\text{A}}_{\text{Sample}} }}{{{\text{A}}_{\text{Blank}} }}\, \times \,100$$

where, A_Blank_: absorbance of the blank reaction, A_Sample_: absorbance of the test compound.

## Conclusion

Summarizing, we may conclude that the presence of electron withdrawing group (*o*-NO2, compound **4**, MIC = 4.5 μM/ml) improved the antibacterial activity against *E. coli* while presence of *p*-Cl, *p*-Br groups, compounds **3** (IC_50_ = 29.04 μg/ml) and **9** (IC_50_ = 27.66 μg/ml) improved the antioxidant activity. The presence of electron releasing groups, **10** (3, 4, 5-trimethoxy, MIC = 4.2 μM/ml) and compound **15** (*o*-OCH_3_, MIC = 2.60 μM/ml) enhanced the antimicrobial activity against *K. pneumonia*, *S. aureus*, *B. subtilis*, *S. typhi*, *C. albicans* and *A. niger.* Compounds **12** and **15** (*p/o*-OCH_3_, IC_50_ = 27.63 and 22.35 μg/ml) exhibited excellent antidiabetic activity. So, these compounds may be used as lead for the development of novel therapeutic agents.
